# The Role of Oxidative Stress and Inflammation in the Pathogenesis and Treatment of Vascular Dementia

**DOI:** 10.3390/cells14080609

**Published:** 2025-04-17

**Authors:** Aseel Y. Altahrawi, Antonisamy William James, Zahoor A. Shah

**Affiliations:** 1 Department of Pharmacology and Experimental Therapeutics, College of Pharmacy and Pharmaceutical Sciences, The University of Toledo, Toledo, OH 43614, USA; 2 Department of Medicinal and Biological Chemistry, College of Pharmacy and Pharmaceutical Sciences, The University of Toledo, Toledo, OH 43614, USA; william.antonisamy@utoledo.edu

**Keywords:** vascular dementia, neuroinflammation, oxidative stress, cerebral hypoperfusion, inflammasome, antioxidant drugs, anti-inflammatory drugs, blood–brain barrier

## Abstract

Vascular dementia (VaD) is a heterogeneous group of brain disorders caused by cerebrovascular pathologies and the second most common cause of dementia, accounting for over 20% of cases and posing an important global health concern. VaD can be caused by cerebral infarction or injury in critical brain regions, including the speech area of the dominant hemisphere or arcuate fasciculus of the dominant hemisphere, leading to notable cognitive impairment. Although the exact causes of dementia remain multifactorial and complex, oxidative stress (reactive oxygen species), neuroinflammation (TNFα, IL-6, and IL-1β), and inflammasomes are considered central mechanisms in its pathology. These conditions contribute to neuronal damage, synaptic dysfunction, and cognitive decline. Thus, antioxidants and anti-inflammatory agents have emerged as potential therapeutic targets in dementia. Recent studies emphasize that cerebrovascular disease plays a dual role: first, as a primary cause of cognitive impairment and then as a contributor to the manifestation of dementia driven by other factors, such as Alzheimer’s disease and other neurodegenerative conditions. This comprehensive review of VaD focuses on molecular mechanisms and their consequences. We provided up-to-date knowledge about epidemiology, pathophysiological mechanisms, and current therapeutic approaches for VaD.

## 1. Introduction

Vascular dementia (VaD) is a major form of dementia that results from vascular brain injury, primarily due to brain parenchyma damage that is associated with ischemia, infarction, or hemorrhage [1]. After Alzheimer’s disease (AD), it is considered the second most prevalent subtype of dementia [2]. As of 2020, there were an estimated 55–60 million patients suffering from VaD worldwide; this number is predicted to be 150–160 million by 2050 [3]. VaD is becoming increasingly common, resulting in a huge economic expense for its treatment, particularly in older people [3]. VaD can develop from global or localized consequences of vascular diseases. However, the most common vascular contribution to dementia is cerebral small-vessel disease (CSVD) [4]. VaD is recognized as a neurocognitive disorder that encompasses behavioral symptoms and locomotor abnormalities such as agitation, aggression, dysarthria, and Parkinson’s-like gait disorder [4].

VaD incorporates various vascular mechanisms and alterations in the brain and has distinct etiologies and clinical manifestations [5]. Growing evidence suggests that cerebrovascular pathology is the primary cause of dementia, with a synergistic interaction with neurodegenerative pathology. Chronic age-related dysregulation of cerebral blood flow (CBF) is the most frequent underlying cause, although other factors such as inflammation, oxidative stress, and cardiovascular dysfunction have a substantial role [6].

One of the pivotal mechanisms implicated in the pathophysiology of VaD is oxidative stress. High peripheral pro-oxidant markers and low antioxidant capacity levels have been documented in patients with VaD [7]. Oxidative stress promotes neuronal damage and vascular injury, worsening the cognitive decline associated with this disease. Elevated levels of reactive oxygen species (ROS) can damage cellular components such as lipids, proteins, and DNA, disturbing neuronal signaling and promoting cell death [8]. Hence, a deep understanding of the role of oxidative stress is crucial for identifying potential targets to decrease the progression of VaD.

Neuroinflammation is another critical factor in the development and progression of dementia. Chronic inflammation, triggered by factors such as hypoxia, ischemia, and infection, promotes the activation of glial cells and the secretion of various proinflammatory cytokines. This process leads to apoptosis, damage to the blood–brain barrier (BBB), and other pathological changes, all of which exacerbate the initiation and progression of VaD [9]. Moreover, several studies have reported high levels of proinflammatory biomarkers in the cerebrospinal fluids (CSFs) and plasma, supporting that neuroinflammation is an important pathological factor of VaD [10,11].

This review summarizes the role of oxidative stress and inflammation in the pathophysiology of VaD. Unlike previous reviews, which focused on specific parts of various underlying mechanisms or therapeutic strategies, this review provides a comprehensive summary of the mechanisms underlying VaD and the majority of agents currently under investigation in preclinical settings. This work will not only fill gaps in the current literature but also provide a major resource for scientists aiming to enhance therapeutic strategies for dementia. Moreover, this review aims to stimulate new research and facilitate the translation of promising agents into therapeutic use for this disastrous disease. Finally, the future potential of targeting endogenous antioxidants and anti-inflammatory biomarkers for preventing the initiation and progression of VaD is highlighted. Relevant studies were searched in Google Scholar and PubMed using the following terms: dementia, ‘vascular dementia’, ‘mild cognitive impairment’, ‘oxidative stress’, ‘antioxidants’, neuroprotective, preclinical, clinical, rats, murine, model, human, and patients. Boolean operators were used to retrieve a focused search on PubMed. Since this work was designed as a narrative review, no specific guidelines or formal criteria were in place for selecting or evaluating studies.

## 2. Vascular Dementia (VaD)

### Definition and Etiology

VaD was initially known as multi-infarct dementia, indicating cumulative tissue loss caused by recurrent ischemic stroke. Early pathological studies suggested that losing 50 to 100 mL of tissue could result in dementia [12]. However, it became apparent over time that multi-infarct dementia was simply one of several subtypes of VaD. Pathological findings from large patient cohorts demonstrated that subcortical VaD, rather than large cortical infarctions, is the predominant form of VaD [13]. Recently, the Vascular Impairment of Cognition Classification Study (VICCS) defined four fundamental types of vascular lesions leading to dementia: post-stroke dementia, multi-infarct dementia, mixed dementia, and subcortical ischemic VaD [14].

The etiology of VaD can mainly be divided into four key pathways: hypoperfusion and hypoxia, BBB dysregulation, disturbances in cerebrospinal fluid (CSF) and interstitial fluid (ISF) drainage, and vascular inflammation. Each of these items is considered to play an independent role in the pathogenesis of VaD [15]. Hypoxia and hypoperfusion can result in persistent oxygen deprivation in brain tissues, especially the white matter, resulting in neuronal damage and cognitive decline [16]. BBB dysregulation leads to plasma protein leakage and leukocyte infiltration into the brain, promoting glial activation, demyelination, and neurodegeneration, eventually resulting in cognitive decline in patients with VaD [17]. Disturbances in CSF and ISF drainage can limit the clearance of neurotoxic waste, contributing to protein accumulation and white matter damage and leading to cognitive impairment [17]. Vascular inflammation causes endothelial dysfunction, oxidative stress, and atherosclerosis, further decreasing CBF and accelerating cognitive decline [17].

In addition to these central risk factors, peripheral modifiable risk factors such as hypertension, hypercholesterolemia, obesity, and diabetes also have a vital role in the development and progression of VaD [18]. These factors exacerbate systemic inflammation by increasing circulating cytokines, which can damage the BBB, activate microglia, and aggravate the central neuroinflammatory response [18]. As described by Zhang et al., this peripheral-to-central immune signaling creates a vicious cycle that perpetuates vascular and neuronal injury [19].

## 3. ROS Contribution to Neuronal Injury

ROS, primarily generated in the mitochondria as a natural byproduct of normal metabolic processes, are involved in cell signaling [20]. The physiological level of ROS plays a critical role in controlling CBF by acting as a potent vasodilator, with studies demonstrating that the generation of NADPH-induced hydrogen peroxide in cerebral vessels leads to vasodilation [21]. Within the vasculature system, ROS are generated from enzymatic and non-enzymatic sources [22]. The superoxide anion is the primary ROS, produced by one-electron reduction of molecular oxygen through the activity of oxidases such as NADPH oxidase, cyclooxygenase, mitochondrial electron transport chain enzyme, lipoxygenase, and cytochrome P450 (CYP 450) enzymes [22]. The superoxide anion is then converted to hydrogen peroxide through the activity of the superoxide dismutase (SOD) enzyme [23]. Disruption of the normal physiological redox balance can have harmful consequences by producing peroxides and free radicals, damaging multiple cellular components such as proteins, lipids, and DNA [24].

In VaD, erroneous electron transport chain (ETC) activity and mitochondrial dynamics contribute to cerebrovascular injury and enhance ROS production [25]. Oxidative phosphorylation (OXPHOS) is a crucial process within the mitochondria that generates adenosine triphosphate (ATP), which is particularly important for energy-demanding cells like neurons [26]. OXPHOS involves the ETC composed of complexes encoded by nuclear and mitochondrial DNA (nDNA and mtDNA). Disruption of the regulation of mtDNA, particularly through the mitochondrial transcription factor A (TFAM), can result in declined mitochondrial bioenergetics [26,27]. Due to the brain’s high metabolic demand and oxygen consumption, it is an important source of ROS as a byproduct of OXPHOS. The generated ROS attacks DNA bases and causes various damages such as DNA strands breaking, DNA–DNA and DNA–protein cross-linking, and oxidized base adducts such as 8 hydroxyguanine (8-OHG). The oxidative damage is most obvious in mtDNA, which lacks protective histone and has few repair mechanisms [28]. The DNA damage can alter protein expression and enzyme activity, eventually affecting neuronal metabolism and contributing to cell death [29].

ROS-induced lipid oxidation can damage cellular membranes, producing toxic secondary products, including 4-hydroxy-2-nonenal (4-HNE) and acrolein, which are harmful to neurons [28]. Thus, oxidative DNA damage plays a crucial role in the progression of VaD and contributes to mitochondrial function. Furthermore, oxidative stress-induced brain mitochondrial dysfunction dysregulates calcium homeostasis, initiating apoptosis and necrotic cell pathways. These events impair the integrity and survival of neurons, contributing to the cognitive decline observed in VaD [30]. Therefore, targeting mitochondrial dysfunction offers a promising therapeutic approach to mitigate neuronal damage and prevent the progression of VaD.

Chronic cerebral hypoperfusion is an essential element in vascular cognitive impairment, leading to BBB dysfunction. Prolonged hypoperfusion creates endothelial stress, which increases BBB permeability by reducing tight junction proteins and damaging the endothelium glycocalyx. This environment stimulates inflammation, as evidenced by microglia and astrocyte activation, as well as the generation of proinflammatory cytokines and chemokines, which further compromise BBB integrity. Furthermore, oxidative stress from ROS exacerbates endothelial damage, resulting in neurovascular uncoupling and impaired cerebral autoregulation [31].

In VaD, hypoperfusion not only increases these harmful processes but also triggers the activation of matrix metalloproteinase enzymes (MMPs), especially MMP-9 and MMP13, which are responsible for degrading the extracellular matrix surrounding the blood vessels. This enzymatic degradation further promotes BBB dysfunction by compromising the structural integrity of brain capillaries [32]. MMP-13, in particular, promotes pericyte detachment, which, along with MMP-9-mediated matrix degeneration, disrupts endothelial pericyte connections and reduces neurovascular unit stability. The disturbances in the BBB occur regardless of amyloid beta (Aβ) or tau levels, aging processes, or vascular risk factors [33]. The resulting injury to the capillary mural cells, particularly pericytes responsible for maintaining BBB integrity, increases BBB permeability, especially in regions essential for memory and cognitive functions. As a consequence, neurotoxic substances penetrate the brain, potentially contributing to neuronal damage, cognitive decline, and the progression of dementia [32,33]. Chronic hypoperfusion is also closely associated with CSVD, which manifests as BBB leakage, white matter lesions, and lacunar infarctions, all of which contribute to the progression of VaD [31].

Beyond that, cerebral hypoperfusion and ischemic events increase the brain’s susceptibility to lipid peroxidation [34], a process where ROS attacks the polyunsaturated fatty acids in cell membranes, resulting in the production of harmful byproducts such as malondialdehyde (MDA), an indicator for oxidative damage [35]. Elevated levels of lipid peroxidation byproducts, including MDA and 4-HNE, were observed in VaD [34]. The brain’s high oxygen demands and abundance of polyunsaturated fatty acids make it particularly vulnerable to lipid peroxidation [35,36]. The resulting damage affects membrane integrity, altering cellular activities and causing cell death, all of which contribute to the progression of VaD [36]. As lipid peroxidation progresses, it exacerbates the neurodegenerative process by disrupting the function of essential proteins and enzymes, such as Na+/K+ ATPase and NMDA receptors [37]. This not only causes neuronal dysfunction but also promotes inflammatory responses that further damage the cerebral endothelium [36]. Recent studies revealed that increased levels of lipid peroxidation products (e.g., MDA and 4-HNE) and reduced antioxidant levels are early indicators of neurodegenerative diseases, including VaD [38,39].

This cascade of lipid peroxidation is amplified by inadequate antioxidant defense mechanisms, resulting in more cognitive damage and neuronal loss [39]. The body’s defense systems, which include natural antioxidants, including SOD, catalase (CAT), and glutathione peroxidase (GPx), help to mitigate the resulting damage. However, any imbalance in these systems worsens oxidative damage, accelerating the progression of VaD [40]. Additionally, a recent meta-analysis found that glutathione (GSH) depletion in VaD, a critical antioxidant that protects against oxidative stress, results in the impairment of cellular redox equilibrium, detoxification mechanisms, mitochondrial dysfunction, and dysregulation of nitric oxide (NO) generation, all of which are essential for maintaining the CBF and vascular integrity [41]. Therefore, targeting the GSH level could be a promising therapeutic strategy for halting these pathological processes in VaD. Collectively, these findings highlight the critical interplay between lipid peroxidation, oxidative stress, and cerebrovascular factors in VaD, emphasizing the need for targeted therapy to mitigate oxidative stress and slow the disease progression.

## 4. Neuroinflammation and VaD Progression

Neuroinflammation is defined as the activation of the brain’s innate immune system when exposed to different inflammatory stimuli [42]. It is characterized by a variety of cellular and molecular alterations inside the brain in which cytokines and other inflammatory mediators play a central role in regulating the process [42,43]. Evidence from experimental and clinical data supports the major role of neuroinflammation in the pathogenesis of all stages of VaD and other neurodegenerative disorders [9,44,45,46]. In VaD, chronic cerebral hypoperfusion activates microglia and astrocytes, producing proinflammatory cytokines such as IL-1β, IL-6, and TNF-α. These cytokines, in combination with oxidative stress, exacerbate neuronal damage, white matter loss, and cognitive decline [9]. Chronic hypoperfusion amplifies the inflammatory response through pathways such as JAK/STAT, TLR4/MYD88, and NF-kB/STAT3, leading to continuous neuroinflammation [47,48,49].

VaD pathophysiology primarily depends on immune cells, such as microglia, astrocytes, oligodendrocytes, and boundary-associated macrophages (BAMs) [9]. Microglia, for example, undergo phenotypic alterations in response to hypoperfusion, with some providing a protective role by secreting growth factors and anti-inflammatory mediators, while others intensify neuroinflammation by releasing proinflammatory cytokines [50]. Astrocytes are also affected, and they are responsible for maintaining the BBB integrity. Chronic hypoperfusion leads to astrocyte activation, pseudopodia fracture, and decreased expression of aquaporin-4 (AQP4), contributing to BBB breakdown and cognitive decline [9].

The inflammatory response in VaD undergoes several phases. Initially, microglia and macrophages clear necrotic debris, but as inflammation progresses, it triggers mitochondrial and DNA damage, causing cell death [9]. Hypoxia exacerbates these outcomes by increasing hypoxia-inducible factors (HIFs), which activate additional inflammatory pathways, causing neuronal loss and impaired neuroplasticity [51,52]. Chronic inflammation, including high levels of inflammatory cytokines such as IL6, TNF-α, and C-reactive protein (CRP), is a driving force in the development and progression of VaD [53]. Among these inflammatory biomarkers, IL-6 stands out. VaD patients exhibit continuously elevated levels of IL-6 in their blood and CSF, differentiating them from both healthy individuals and those with AD [54]. A recent meta-analysis found that IL-6 levels were significantly greater in VaD patients than in AD individuals, highlighting IL-6 as a potential diagnostic marker and predictor for disease progression [55]. While C-reactive protein (CRP) association with VaD is less clear, studies proved that elevated CRP levels combined with high IL-6 dramatically increase the risk of VaD [55]. TNF-α, a crucial cytokine, has been implicated in the pathogenesis of VaD, with few studies reporting elevated levels in VaD patients compared to healthy controls [55].

In addition to these cytokines, inflammasomes such as NLRP3 and AIM2 play an important role in controlling chronic inflammation. Under hypoxic conditions, their activation triggers the production of IL-1β and IL-18, leading to disruption of the BBB, oligodendrocyte loss, and damage to the neurovascular unit, all of which have been linked to cognitive decline in VaD [56]. The complement system also exacerbates neuroinflammation, causing neuronal and vascular damage [57]. In VaD, the activation of the complement system, mainly through the classical and lectin pathways, produces membrane attack complex (MAC) and releases proinflammatory cytokines, which can further damage the BBB [58].

The impact of the complement system on VaD is primarily mediated by its complicated interaction with other physiological systems. For instance, its interaction with the coagulation cascade promotes a prothrombotic state, which contributes to microvascular occlusion that reduces CBF and worsens cognitive impairment [59]. On the other hand, chronic complement activation, in response to infectious pathogens, can potentially promote neurodegeneration by triggering inflammation and the accumulation of neurotoxic proteins such as Aβ [60]. This chronic activation links infectious diseases to the development and progression of different neurodegenerative disorders, including VaD [60]. Global complement suppression might be a promising strategy to treat VaD by mitigating inflammation and thrombotic risk; however, careful modulation is necessary to prevent impairing normal immunological and hemostatic function.

**Figure 1:**
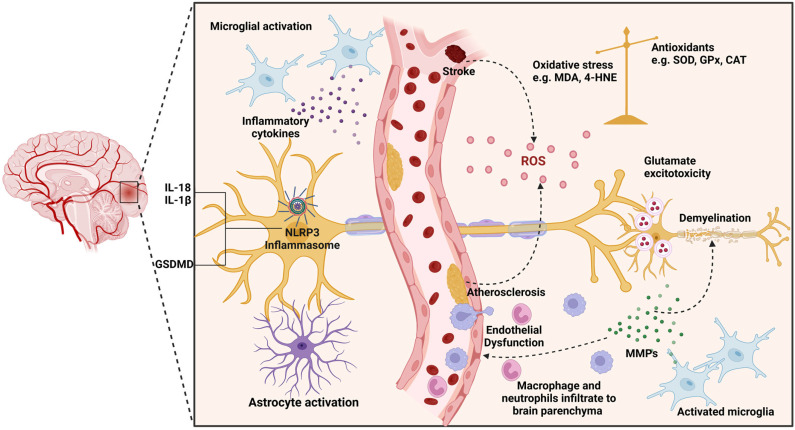
Mechanism of Vascular Dementia. Ischemic stroke occurs when a clot in the cerebrovascular system leads to reduced blood flow (hypoperfusion) and tissue oxygen deprivation (hypoxia). This lack of oxygen activates glial cells, which then produce pro-oxidants, reactive oxygen species (ROS), proinflammatory cytokines, and matrix metalloproteinases (MMPs) from activated microglia, reactive astrocytes, and oligodendrocyte progenitor cells (OPCs). The ROS causes DNA damage through oxidative stress, leading to neuronal death. Inflammatory cytokines induce neuroinflammation and further neuronal death, disrupt the blood–brain barrier (BBB), and promote the expression of adhesion molecules in endothelial cells, facilitating the infiltration of leukocytes and platelet adhesion and microvascular occlusion.

Another critical factor in the progression of VaD is the activity of matrix metalloproteinases (MMPs). MMPs, known for their ability to break down extracellular matrix (ECM) components, play an important role in vascular remodeling and BBB disruption, key components in the pathophysiology of VaD [61]. MMPs cause neuroinflammation by degrading myelin and loosening tight junctions in endothelial cells, both of which are exacerbated by chronic cerebral hypoperfusion [62]. This series of events contributes to what is known as “by-stander demyelination”, a characteristic finding of the cognitive decline observed in VaD [62]. MMP’s detrimental effects in VaD are confirmed by different studies that linked them to BBB permeability and inflammatory response. For example, several studies have shown that higher levels of MMP-2 and MMP-9 are associated with the disruption of the BBB and degradation of the ECM, both of which are important contributors to the development of white matter injuries in VaD [63,64]. Figure 1 summarizes the possible mechanisms involved in the pathogenesis of VaD.

## 5. Animal Model to Induce VaD

The development of a valid animal model that precisely replicates the pathophysiology and outcomes of human VaD is a crucial initial step toward investigating potential therapeutic options to slow the progression of the disease [65]. Currently, several animal models have been established to study VaD [66]. However, when choosing a VaD model, it is important to recognize that VaD can occur from various pathophysiological mechanisms and risk factors, individually or in combination [65]. Among the most widely used models for investigating VaD are the murine model of global ischemia, models of chronic cerebral hypoperfusion induced by bilateral carotid artery occlusion/stenosis, and SVD resulting from high blood pressure in spontaneously hypertensive rats [66].

The standard animal model of VaD involves permanent blocking of both common carotid arteries. This method aims to disrupt energy production in neurons and glial cells, accelerating the synthesis of amyloid protein and thus resulting in dementia [67]. This model is commonly used to study post-stroke dementia as it simulates the acute ischemic injury and subsequent cognitive deterioration that occurs post-cerebral vascular events. For instance, in a preclinical study conducted by Poh et al., the impact of chronic cerebral hypoperfusion was studied using bilateral common carotid artery stenosis (BCAS) in mice. The results revealed significant cognitive impairment, as observed by poor performance in the Morris Water Maze (MWZ). The neuropathological investigation demonstrated widespread white matter loss, impaired myelin integrity, and hippocampus cell death, especially in CA1 and CA2 regions. Moreover, there was a significant increase in inflammasome signaling with elevated NLRP3, AIM2, and NLRC4 expression in the cerebral cortex. This activation was associated with increased caspase-1, IL-18, and IL-1β production, reflecting a significant inflammatory response. BCAS also increased apoptosis (observed by increased levels of caspase-3) and pyroptosis (a high level of gasdermin D was seen in the BCAS group) in the brain and hippocampus, with greater apoptosis noticed in the striatum [68]. Washida et al. developed a baboon model of vascular cognitive impairment using a three-vessel occlusion (3-VO), in which they occluded both internal carotid arteries and the left vertebral artery. Two weeks after the surgery, the animals developed demyelination in the white matter, which is consistent with other animal models of cerebral hypoperfusion. Although the grey matter lesions were rare, the baboons experienced temporary hemiparesis, partial paralysis on one side of the body, after the procedures [69].

The middle cerebral artery occlusion (MCAO) murine model is commonly used to study the effect of ischemic stroke, a key contributor to VaD, in humans. In this model, a transient obstruction of the middle cerebral artery leads to focal cerebral ischemia, which results in infarction in the cortex and striatum [70]. MCAO represents both subtypes of VaD as it contains both CSVD-related changes and global hypoperfusion, together leading to ischemia damage and cognitive impairment. For example, Wang et al. revealed that rats exposed to MCAO experienced severe brain injury as evidenced by an increase in neurological scores and infarct volume. Compared to the sham group, MCAO rats had a significant cognitive deficit, indicated by lower performance in the MWM test. Histopathological findings demonstrated a substantial neuronal loss in the ischemic cortex, increased apoptosis as seen by elevated caspase-3 and Bax, and decreased expression of antiapoptotic Bcl2. In both serum and brain tissue, inflammatory cytokine levels were considerably altered with increased IL-6 and TNF-α and decreased levels of anti-inflammatory mediators like IL-10 and TGF-β1. Furthermore, MCAO enhanced autophagy, which was observed by higher expression of autophagy-related markers (ATG-5, Beclin1, and LC3) and decreased P62 level [71].

The spontaneously hypertensive rat (SHR) model is commonly used to investigate the effect of chronic hypertension on cognitive impairment and VaD, as it reflects the neuropathological impact of high blood pressure on brain functions and structure in a manner similar to that observed in humans [72,73]. This model primarily represents VaD associated with CSVD, a common subtype characterized by chronic hypertension and subcortical ischemic lesions. Sabbatini et al. used SHRs to evaluate the effect of chronic hypertension on the hippocampus. In this model, rats developed hypertension as they aged, with systolic blood pressure substantially higher than the wild-type normotensive rats. The results showed that a significant hippocampus alteration started to appear at age 4 months, with a reduction in white matter volume in the CA1 region and the dentate gyrus. At 6 months of age, there was an additional loss in white and grey matter in the CA1 region and a significant decrease in neurofilament staining reflecting axonal damage. Moreover, SHR had an increase in the glial fibrillary acidic protein (GFAP) expression due to increased astrocyte number, “astroglial hyperplasia”, which indicates an inflammatory response to neuronal damage. However, dendritic integrity was not affected by high blood pressure, suggesting that hypertension produced axonal rather than dendritic injury in the hippocampus [74].

Moss and Jonak established a primate model of VaD by introducing chronic hypertension in middle-aged cynomolgus monkeys using deoxycorticosterone acetate (DOCA) and a high-salt diet for two years. These monkeys demonstrated progressive cognitive deficits, particularly in working memory and cognitive flexibility, which closely resembles the cognitive profile of human subcortical ischemic VaD, which is primarily caused by disturbances in frontal–subcortical circuits. Furthermore, neuropathological findings, including arteriolar thickening, microinfarcts, white matter damage, and increased expression of oxidative stress and inflammatory markers, are consistent with the hallmark alteration observed in human VaD [75]. The anatomical and functional similarities between human brains and these primates increase the model’s translational ability to study different disease mechanisms and test potential interventions to treat VaD.

## 6. Interventions Targeting Oxidative Stress and Inflammation in VaD

### 6.1. Use of Antioxidants and Anti-Inflammatory Agents in Preclinical Settings

Given that the pathogenesis of VaD included increased ROS production and inflammation, it was reasonable to evaluate whether antioxidants or anti-inflammatory agents can reduce the risk of VaD. Several experimental studies that targeted oxidative stress producers, scavengers, and proinflammatory biomarkers or used exogenous antioxidants and anti-inflammatory agents to prevent the incidence and progression of VaD are summarized in Table 1.

### 6.2. Shortcomings of Anti-Inflammatories and Antioxidant Drugs

Despite the promising preclinical results, the tested drugs have shown several drawbacks that prevent moving these drugs forward into clinical settings. This is mainly because inflammation and oxidative stress are crucial factors in many diseases, but their exact mechanism in VaD is not fully understood [8,76]. This makes it difficult to create treatments that target these pathways without unintended consequences. Also, there is limited evidence supporting the long-term benefits of these drugs in treating VaD [77]. Many studies on anti-inflammatory and antioxidative treatments are short-term (5 to 6 months), and the long-term safety and benefits of these interventions in VaD have not been validated [77].

One major concern affecting the translational potential of these drugs in clinical settings is their efficacy. For example, a large number of anti-inflammatory and antioxidant drugs have been clinically tested to treat different neurodegenerative disorders. The anti-inflammatory drugs have been inconsistent in slowing disease progression or improving cognitive function [78,79], while the antioxidants may not always prevent oxidative damage or improve symptoms, especially when the disease pathogenesis mechanisms are complex [80].

Another concern is related to drug toxicity and adverse drug events; chronic use of anti-inflammatory drugs, especially those affecting the cyclooxygenase (COX), can lead to cardiological issues, nephrotoxicity, and GI problems, especially in elderly people, who are prone to develop VaD [81,82]. Similarly, chronic use of antioxidants can be associated with disrupting normal cellular processes, ultimately causing toxicity or other health issues [83].

In clinical practice, many VaD patients have other comorbidities, and they are prescribed multiple medications. Both antioxidants and anti-inflammatory drugs interact with other drugs, particularly those used for chronic conditions, potentially reducing the effectiveness of other medications [84,85]. Further, most anti-inflammatory agents interact with blood thinners and antihypertensive medications, increasing the incidence of adverse drug events [81,86]. Furthermore, clinical practices focus on managing the symptoms and underlying conditions that contribute to the progression of the disease rather than managing the CSVD itself. The anti-inflammatory and antioxidative treatments may reduce the symptoms of dementia, and this could lead to a false sense of improvement without real long-term benefits.

### 6.3. Use of Antioxidants and Anti-Inflammatory Agents in Clinical Settings

Despite the large number of agents that were preclinically tested for VaD (as summarized in the previous table), only a few agents have been clinically tested. For this section, selected studies were searched in PubMed using the clinical trial filter in the advanced search. The search was limited to articles published between 2015 and 2024.

Ginkgo biloba is one of the most popular herbal remedies used to treat memory problems associated with aging [87]. In fact, elderly people frequently use the standardized GB extract (EGb 761) as a dietary supplement to improve memory and prevent cognitive decline [88]. EGb 761 has demonstrated a protective effect against neuronal and vascular injury associated with VaD [89]. For instance, a systematic review conducted in 2016 proved that EGb 761 can effectively improve cognitive levels and mitigate the psychological symptoms associated with VaD [90]. Moreover, a 12-month longitudinal retrospective study of 77 patients with MCI assessed the efficacy of EGb761 alone or in combination with AChEI for treating VaD and found that EGb 761 alone or in combination with AChEI exhibited significant cognitive and behavioral symptoms’ improvement compared to AChEI alone. The combination showed the greatest improvement, significantly enhancing memory, cognitive, and executive functions compared to monotherapies [91]. Likewise, a phase IV, single-center, randomized, open-label clinical trial was also aimed at investigating the effect of EGb 761 extract on the inflammatory and oxidative stress biomarkers as well as the cognitive function in patients with MCI. One hundred participants were randomized in a 1:1 ratio to receive either EGb 761 (240 mg PO once daily) or no therapy (a control group) for 1 year. The primary outcome was changes in the blood levels of inflammatory (VEGF, IL-6, IL-1β, INF-γ, and TNF-α) and oxidative stress biomarkers (GDNF, GFD, SOD2, catalase, and GGT1), while the secondary outcome was the assessment of the cognitive and neuropsychiatric symptoms. The study found that EGb 761 significantly lessened the inflammatory and oxidative stress biomarkers and improved cognitive performance over the treatment period [92]. These results support the preclinical findings that Ginkgo biloba extract (BB) has the ability to reduce oxidative stress biomarkers [93]. Moreover, preclinical and clinical studies demonstrated that Ginkgo biloba extract improves cognitive performance, suggesting a translational effect from animal models to human applications. Collectively, the results of these studies suggest that EGb 761 has the potential to mitigate cognitive decline in MCI patients by modulating inflammation and oxidative stress.

Glucosamine is another naturally occurring substance that has been proven to have anti-inflammatory and antioxidant effects [94,95]. Glucosamine is a naturally occurring amino monosaccharide that serves as a building block for glycosaminoglycans, which are important components of cartilage and synovial fluids. It is well known for its role in joint health and is frequently used as a dietary supplement to treat osteoarthritis symptoms [96]. An observational cross-sectional study found an association between glucosamine consumption and better cognitive performance [97]. Based on this association, Zheng et al. conducted a longitudinal observational study that included 494,814 participants without baseline dementia and followed them for 8.9 years. Regular glucosamine consumption was associated with a lower risk of all-cause dementia, including VaD (HR = 0.74, 95% CI = 0.58–0.95). The Mendelian randomization (MR) analyses also suggest a causal relationship between glucosamine use and dementia risk [98].

Butylphthalide, a chemical compound originally derived from Chinese celery, has been found to have a neuroprotective effect in ischemic stroke via anti-inflammatory, antioxidant, and microcirculatory mechanisms [99]. Since ischemic stroke is a leading cause of VaD, several studies were conducted to evaluate its potential neuroprotective effect in VaD. For example, a clinical comparative study evaluated the efficacy of butylphthalide combined with idebenone in treating VaD. Patients, 88 in number, were randomly allocated in a 1:1 ratio to receive either idebenone alone, a well-known compound initially developed to treat AD and other cognitive defects [100], as a control group, or butylphthalide with idebenone (the observational group) for three months. The combination therapy significantly improved cognitive function, decreased serum inflammatory cytokines, and improved vascular endothelial function compared to idebenone alone. Furthermore, both treatments were well tolerated, with no variation in the adverse effects [101]. In line with these findings, Zhang et al. found that combination therapy significantly improved cognitive functions and reduced both inflammatory and oxidative stress biomarkers, with minimal increment in the incidence of adverse events [102]. Together, these findings suggest that combining butylphthalide with idebenone might be a safe and effective approach to improving cognitive function and decreasing neuroinflammation in VaD patients.

Isosorbide mononitrate (ISMN) and cilostazol are two vasodilators that have a promising effect on improving the function of small blood vessels in delivering oxygen to the brain and facilitating the repair of neural connections post-stroke [103,104]. The Lacunar Intervention Trial-1 (LACI-1) was a phase 2a dose-escalation study designed to evaluate the safety, tolerability, and mechanistic effect of ISMN with cilostazol alone or in combination in patients who experienced lacunar ischemic stroke. The study included 60 patients who were randomly assigned into four groups: cilostazol alone, ISMN alone, a combination with immediate start, and a combination with delayed start. Results demonstrated that both drugs alone or in combination are well-tolerated and have promising effects on cerebrovascular functions [105]. Based on these findings, the LACI-2 trial, a larger phase 2 trial, evaluated the safety and efficacy of ISMN combined with cilostazol in preventing the progression of SVD. The study found that ISMN significantly decreased recurrent stroke and improved cognitive functions, while cilostazol reduced functional dependency. Additionally, combination therapy provided the greatest benefits, reducing the composite outcomes of vascular events, dependency, and cognitive impairment and improving patients’ quality of life [104].

Sildenafil, another phosphodiesterase (PDE-5) inhibitor, can increase CBF by increasing the intracellular level of cGMP, resulting in vasodilation [106]. The neuroprotective effect of sildenafil has been established in different animal models of brain disorders by improving cognitive function, restoring neuronal growth, and alleviating brain injury [107,108,109]. Therefore, the OxHARP trial, a randomized, double-blinded, crossover, placebo-controlled study, evaluated the effect of sildenafil on cerebrovascular function in patients with CSVD. The study included 75 patients with mild to moderate white matter hyperintensities (WMHs) who were randomly assigned to a three-phase crossover trial and received Sildenafil 50 mg TID, cilostazol 100 mg BID, and a placebo. Sildenafil did not appear to decrease the middle cerebral artery pulsatility (a primary endpoint), but it significantly increased the cerebrospinal reactivity and cerebral perfusion. Moreover, sildenafil improved cerebrovascular resistance and increased CBF, indicating more tolerability compared to cilostazol, which caused more adverse effects, including diarrhea. These results suggest sildenafil as a potential treatment for targeting stroke prevention and cognitive decline [110].

**Table 1:** Summary of preclinical studies using antioxidative stress and anti-inflammatory treatment.

Agent	Animal Model	Drug Protocol	Target/Pathway	Main Findings	Ref.
Banhabaekchulcheonma-Tang (BBCT)	BCAS, C57BL/6 mice	80 mL/kg and 40 mL/kg PO, three times a week for 6 weeks starting 2 weeks after surgery	N/A	BBCT treatment significantly enhanced BCAS-associated memory impairment. It decreased microglia and astrocyte activation and reversed BCAS-dysregulated gene expression. BCCT exerts its neuroprotective effect by modulating neuropeptide signaling, promoting neuronal survival, and synaptic stability.	[111]
Berberine chloride	Permanent BCCAo, Wister rats	50 mg/kg PO, daily for 2 months	N/A	Berberine administration increases spatial learning, mitigates memory impairment, alleviates histological damage, and suppresses AchE activity, improving cholinergic function. Additionally, it reduces apoptosis and necrosis in the CA1 region of the hippocampus.	[112]
Betulinic acid	Permanent BCCAo, Wistar rats	10 and 15 mg/kg, PO, once daily starting from D8 to D30 after surgery	N/A	BA has a neuroprotective effect against memory impairment and neuroinflammation induced by VaD. It significantly improved cerebral blood flow after BCCAO reduced proinflammatory and oxidative stress markers and restored cAMP, cGMP, and neurotransmitters (e.g., DA, NE, and 5-HT3) to levels comparable to those in the sham group.	[113]
Bilobalide (Ginkgo biloba extract)	2-VO, SD rats	2, 4, and 8 mg/kg, IGAS for 2 months	N/A	BB treatment significantly alleviated memory and learning impairment associated with 2-VO. BB reduces oxidative stress (decreasing the MDA level and NOS activity and increasing the SOD activity and GSH level). It also mitigates neuronal histological changes associated with vascular insult in both the hippocampus and cortex.	[93]
Calmodulin inhibitor (DY-9836)	BCAS, C57BL/6 mice	0.5 or 1 mg/kg, PO from D 5 to D 45 post-operation	NLRP3 inflammasome pathway	Repeated administration of DY-9836 attenuated BCAS-induced learning and cognitive impairment. It restored the phosphorylated CaMK-II in hippocampal CA1 neurons and reduced inflammation by inhibiting the NLRP3/Caspase-1/IL-1β signaling pathway.	[114]
Carnosine	BCCAO (alternate cycles of occlusion/relaxation of 10 min each for three cycles), Wistar rats	200 or 400 mg/kg, IP once daily for 9 days after surgery	N/A	Carnosine administration mitigates CCH-induced spatial and cognitive impairment. It reduced oxidative stress by decreasing oxidative damage markers (e.g., AchE activity, MPO activity, and TBAR level) and increasing GSH levels. Its anti-inflammatory effect was observed by decreasing the neutrophil filtration.	[115]
Cilostazol and simvastatin	L-methionine-induced VaD, Wistar rats	Simvastatin (50 mg/kg, PO) or cilostazol (100 mg/kg, PO), respectively, for 32 days	N/A	Both drugs alleviate L-Met-induced memory impairment by decreasing oxidative stress and inflammation. Simvastatin and cilostazol attenuate AchE activity and increase brain endothelial nitric oxide synthase levels, reducing amyloid beta-42 and cholesterol levels.	[116,117]
Citicoline and Nicotinamide	Permanent BCCAO, SD rats	Citicoline (160 mg/kg) and Nicotinamide (40 mg/kg), IP once daily for 4 Weeks, started 1 week after surgery	SIRT1/TORC1/CREB pathway	Citicoline and NMN synergistically attenuate BCCAO-induced white matter damage and cognitive impairment by activating the SIRT1/TORC1/CREB pathway. They inhibit microglia activations, reduce proinflammatory cytokines (IL-1β, IL-6, and TNF-α), and increase anti-inflammatory mediators (e.g., IL-10). These findings highlight the neuroprotective effect of this combination.	[118]
*Clostridium butyricum* (probiotics)	permanent right unilateral common carotid artery occlusion (rUCCAO), ICR mice	(1 × 10⁶, 1 × 10⁷, and 1 × 10⁸ CFU/mL), 200 mcl IGAS daily for 6 weeks	BDNF-PI3K/Akt pathway	*C. butyricum* improved cognitive performance and decreased neuronal death in VaD by modulating the gut–brain axis and enhancing butyrate levels in the brain, ultimately activating the BDNF-PI3K/Akt signaling pathway.	[119]
Co-ultraPEALut (palmitoylethanolamide + luteolin)	BCCAO (alternate cycles of ligation/relaxation of 10 min each for three cycles), CD1 mice	1 mg/kg, PO daily for 15 days	NF-κB	Co-ultraPEALut attenuates BCCAo-induced memory impairment. It inhibits the NF-κB activation by blocking IκB-α degradation, reducing proinflammatory markers (COX-2, iNOS), and decreasing oxidative stress (nitrotyrosine production). It inhibited apoptosis by decreasing Bax and increasing Bcl2 expression. It also exerts a neuroprotection effect on hippocampal neurons by increasing BDNF and NT-3 expression.	[120]
Danggui-Shaoyao San	Permanent BCCAo, SD rats	(1.8 g/kg or 7 g/kg) once daily for 4 weeks	IKK/NF-κB	DSS attenuated cognitive dysfunction in VaD rats induced by BCCAO, as observed by attenuating memory deficits, alleviating neuronal apoptosis through regulating the Bcl-2/Bax ratio, cleaved caspase-3, and oxidative stress pathways, and reducing oxidative stress markers (MDA, ROS). DSS has an anti-inflammatory effect, as shown by reducing TNF-α and IL-1β.	[121]
Danshen–Honghua Herbal Pair	Permanent BCCAO, Wistar rats	3.2 g/kg/day, IGAS, for 4 weeks	N/A	DH enhanced cognitive performance in VaD rats. It also restored the cholinergic balance by increasing the Ach level and decreasing the AchE enzyme activity. It has a protective effect against neuronal apoptosis, as demonstrated by decreasing the ROS accumulation.	[122]
Dimethyl fumarate (DMF)	MCAO, SD rats	12.5 mg/kg PO twice daily for 3 days before and 10 consecutive days after surgery	N/A	DMF treatment mitigated oxidative damage, oxidative stress, and neuroinflammation associated with post-stroke cognitive impairment. It improved cognitive function and reduced oxidative stress, apoptosis, and autophagosome formation in the hippocampal CA1 region.	[123]
Duloxetine	Permanent BCCAO, SD rats	20 mg/kg, IP, once daily for 4 weeks	mTOR/S6K	DXT treatment protects against CCH-induced hippocampal neuronal damage in the CA1 region. Its neuroprotective effect is mediated by maintaining the TOR/S6K signaling pathway. DXT treatment also decreased proinflammatory biomarkers (e.g., TNF-α, IL-1β).	[124]
Edaravone	Permanent BCCAO, Wistar rats	3, 5, and 6 mg/kg or IP for 28 days after surgery	ERK/Nrf2/HO-1	Edaravone treatment significantly attenuates CCH-induced spatial and fear memory impairment. It reduces oxidative stress by enhancing the activity of antioxidants (e.g., SOD, HO-1) and decreasing the level of oxidative stress markers (e.g., MDA, LDH, ROS). Moreover, edaravone restored synaptic integrity by increasing the production of key hippocampal proteins and improving the phosphorylation of others critical for memory-related signals.	[125,126]
Edible bird’s nest (EBN)	Permanent BCCAO, SD rats	60 mg/kg, 120 mg/kg PO, OD 8 weeks	N/A	Chronic EBN treatment attenuates CCH-induced cognitive impairment and pathological alteration in hippocampal neuronal cells. It preserved neuronal cell viability and reduced oxidative stress and neuroinflammation in the hippocampus, suggesting its potential as a neuroprotective drug to slow the progression of VaD.	[127]
Estrogen	Permanent BCCAO, SD rats	17β-estradiol 100 µg/kg/day, IP for 8 weeks	Wnt/β-catenin pathway	Estrogen treatment significantly ameliorates cognitive damage and neuronal destruction. It inhibits autophagy by reducing the expression of Beclin-3 and LC3B. Furthermore, estrogen activates the Wnt/β-catenin pathway as indicated by increased expression of β-catenin and cyclin-D while decreasing the synthesis of glycogen kinase 3β.	[128]
Genistein	BCCAo (30 min), mice	5.0 and 10 mg/kg PO, OD for 30 days	N/A	Genistein enhanced cognitive function, reduced neuronal apoptosis, and enhanced cellular viability in the CA1 region of the hippocampus. It also increased glucagon-like peptide-1 levels and inhibited dipeptidyl peptidase-4 activity.	[129]
GJ-4	Permanent BCAS, mice	50 mg/kg, IGAS, 4 weeks	Keap1-Nrf2/HO-1 pathway	GJ-4 improved both short- and long-term memory. It has a neuroprotective effect against VaD mediated by decreased oxidative stress through the Keap1-Nrf2/HO-1 pathway, enhancing lipid metabolism. Moreover, it has an antiapoptotic effect, seen by increasing the Bcl2/Bax ratio. GJ-4 also enhanced remyelination as indicated by increased expression of myelin-related protein (e.g., MBP, MOG, and MAG), which is essential for cognitive function improvement.	[130]
Ling-Yang-Gou-Teng-decoction (LG)	Autologous microthrombi injection against the background of hypercholesterolemia induced with a high fatty diet, Wistar rats	(2.58, 8.14, 25.80 g/ kg/day) PO, 3 days before and 3 weeks after microthrombi injection	N/A	Repeated LG exposure significantly enhanced cognitive function and memory ability. LG exerts its antioxidant effect by decreasing NOX2, a major source of oxidation in VaD, and upregulating the expression of SOD3. Moreover, LG mitigated the vascular and neural edema and increased neuronal hippocampus survival.	[131]
Melatonin	Permanent BCCAO, SD rats	20 mcg/mL of melatonin in drinking water for 4 weeks	N/A	Chronic administration of the neurohormone melatonin alleviates VaD-associated neuronal damage by mitigating oxidative stress, illustrated by reducing oxidative stress markers (TBARS) and enhancing the antioxidant activity (SOD, CAT, GSH). Melatonin also decreases the apoptosis in the hippocampal CA1 neurons (modulates the Bcl-2/Bax ratio).	[132]
Osthole	Permanent BCCAo, SD rats	5, 10, and 20 mg/kg PO daily for 62 days	NLRP3 inflammasome pathway	Chronic treatment of osthole alleviates cognitive impairment caused by BCCAo-induced VaD. It has an anti-inflammatory effect, indicated by decreasing microglial activation and downregulating NLRP3 pathway activation. Osthole also decreased Aβ deposition and reduced the expression of APP and BACE1.	[133]
Paeoniflorin	4-VO, SD rats	40 mg/kg IP once daily for 4 weeks	mTOR/NF-κB and PI3K/Akt pathways	PF mitigated cognitive impairment, decreased proinflammatory biomarkers (IL-1β, IL-6, and TNF-α), and increased anti-inflammatory markers (e.g., IL-10 and TGF-β) in the hippocampus. It also protected against morphological damage of hippocampal neurons by activating the PI3K/Akt pathway, which shifts microglial polarization to the M2 phenotype.	[134]
20 (S)—Protopanaxadiol (PPD)	Permanent BCCAo, SD rats	PPD- H, M, L; 20, 10, 5 mg/kg, respectively, SQ, once daily for 3 weeks	NLRP3 inflammasome pathway	PPD exerts neuroprotective effects in VaD rats mainly due to its anti-inflammatory effect, which is mediated by reducing NLRP3 inflammasome activation, preventing amyloid-beta precipitation, and tau phosphorylation.	[67]
Resveratrol	Permanent BCCAO, Wistar rats	25 mg/kg PO, daily for 4 weeks from W8 to W12 after surgery	N/A	Resveratrol ameliorates CCH-induced spatial memory impairment. It significantly attenuates the progression of VaD by reducing the apoptosis, as indicated by decreasing the Bax/Bcl2 ratio and reducing the c-caspase3 and c-PARP protein expression.	[135]
Resveratrol-loaded solid lipid nanoparticles (R-SLNs)	Permanent BCCAO, SD rats	10 mg/kg, PO once daily, starting from W4 to W8 after surgery	Nrf2/HO-1 pathway	R-SLN supplementation provided a neuroprotective effect against VaD. It improved spatial memory retention, mitigated CCH-induced oxidative stress in brain tissue as demonstrated by decreased levels of oxidative stress markers (e.g., MDA, lipid peroxidation, protein carbonyl, and GSSG), and increased the level/activity of antioxidants (HO-1, NRF-2, SOD, and GSH).	[136]
Tetrandrine	2-VO, SD rats	10 mg/kg or 30 mg/kg, IP, Q.o.d for 4 weeks	N/A	Tetrandrine enhanced cognitive performance and attenuated 2-VO-associated hippocampal neuronal necrosis. It decreased IL-1β levels and reduced NR2B phosphorylation.	[137]
Vildagliptin	Pancreatectomy-induced VaD, Wistar rats	3 and 6 mg/kg PO, daily for 2 months		Chronic vildagliptin administration significantly attenuated diabetes-induced memory and executive functioning impairment. Vildagliptin also restored endothelial function and reduced oxidative stress (decreased TBARS and MPO and increased GSH). Moreover, vildagliptin improved BBB integrity and reduced brain calcium levels.	[138]
Zafirlukast, piracetam, and their combination	L-Met-induced VaD, Wistar rats	Zafirlukast (20 mg/kg, PO), piracetam (600 mg/kg, PO), or combination (zafirlukast 20 mg/kg + piracetam 600 mg/kg, PO) OD for 32 days	N/A	Both agents, alone and in combination, improved the behavioral and neurochemical alteration associated with L-Met administration. Zafirlukast and piracetam decreased norepinephrine, dopamine, and Aβ-42 levels; increased Ach levels; and decreased AchE activity. Both drugs enhanced GSH and IL-10 content and reduced IL-6 and MDA levels.	[139]

## 7. The Future of Antioxidants and Anti-Inflammatories as a Therapeutic Target in VaD

Improving endogenous antioxidant and anti-inflammatory capacities in VaD is a crucial therapeutic strategy to mitigate the progression of the disease. This approach focuses on enhancing the natural defense systems to combat oxidative stress and chronic inflammation, which play crucial roles in VaD pathophysiology.

**Figure 2:**
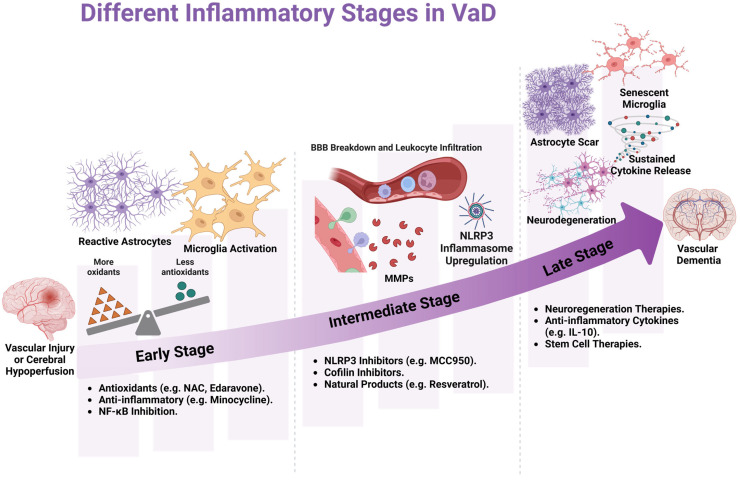
Different stages of inflammation in vascular dementia. In the early stage, vascular injury or cerebral hypoperfusion activates the microglia and astrocytes, exacerbating neuroinflammation and possibly affecting antioxidant system homeostasis. Minocycline and NF-kB inhibitors could be therapeutic agents, and NAC and Edaravone could be antioxidant agents. In the intermediate stage, inflammatory mediators influence inflammasome formation and induce BBB disruption and leukocyte infiltration. It further leads to releases of the MMPs, which induce endothelial cell damage. These conditions lead to astrocyte scar and senescent microglia with sustained cytokine release, contributing to neurodegeneration and dementia at the late stage of inflammation. Neurogenerative therapies, anti-inflammatory cytokines, and stem cell therapies could be potential treatment options.

Activating the endogenous antioxidant defense can inhibit oxidative stress, mitigate neuronal damage, and reduce disease progression. Therapeutic interventions aimed at increasing the expression or activity of antioxidant enzymes (SOD, CAT, and GPx) could reduce oxidative damage in VaD. Genetic or pharmacological agents activating the Nrf2 pathway are promising strategies for enhancing the endogenous antioxidant response. Targeting glutathione (GSH) or its precursor N-acetylcysteine (NAC): Glutathione, one of the most important intracellular antioxidants, is often depleted in VaD. Restoring GSH levels through pharmacological agents could maintain redox balance and protect against mitochondrial dysfunction. Targeting Coenzyme Q10: This improves mitochondrial bioenergetics and mitochondrial functions (stabilizing mitochondrial membranes or enhancing mitophagy and mitochondrial DNA repair) and could mitigate oxidative stress and maintain neuronal health. Chronic neuroinflammation is a key driver of cognitive decline in VaD. Anti-inflammatory interventions are crucial for preserving brain function. Several approaches to improve endogenous anti-inflammatory mechanisms include modulating inflammatory pathways: Targeting the pathways responsible for the production of proinflammatory cytokines, such as JAK/STAT, NF-kB, or TLR4/MYD88 pathways, could mitigate the excessive inflammation while preserving the protective roles of immune function, which is critical to avoid adverse side effects. These biomarkers could also serve as therapeutic targets for personalized medicine approaches. Targeting the inflammasome pathway: Inhibiting NLRP3 could reduce the production of proinflammatory cytokines and limit the damage to the neurovascular unit. Small molecules that suppress inflammasome activation could restore the integrity of the BBB and protect against neuronal injury. Targeting microglial activation or shifting them toward a more anti-inflammatory (M2) phenotype could reduce the neuroinflammation in VaD. Therapies promoting anti-inflammatory cytokines (such as IL-10) or growth factors from microglia while inhibiting proinflammatory cytokine (TNFα, IL1β, and IL-6) release could promote neuroprotection. Combination therapies: Given the multifaceted nature of VaD, combination therapies that simultaneously target oxidative stress, mitochondrial dysfunction, and inflammation should be explored. Such strategies may be more effective in addressing the overlapping pathways involved in VaD progression. Figure 2 summarizes the potential use of these agents at various stages of VaD.

In addition to the potential benefits of targeting these key pathological mechanisms, stem cell transplantation is considered a promising alternative strategy. Recent evidence demonstrated that it improved symptoms of cerebral infarction and Alzheimer’s disease. It aims to restore cognitive function by stimulating neurogenesis and preventing neuronal apoptosis [140]. While animal studies have shown promising results, a single human trial found that stem cell transplantation can only temporarily ameliorate the clinical symptoms of VaD patients [141]. By focusing on these future directions, researchers and clinicians can work toward more effective treatments that address the complex mechanisms underlying VaD, ultimately improving patient outcomes and reducing the global burden of the disease.

## 8. Conclusions

This review provides a comprehensive evaluation of oxidative stress and neuroinflammation as pivotal mechanisms in the pathogenesis of VaD. The roles of ROS in neuronal damage, mitochondrial dysfunction, and BBB disruption have been highlighted as central contributors to cognitive decline in VaD. Likewise, chronic neuroinflammation, mediated by cytokines, inflammasomes, and the complement system, exacerbates the damage to neuronal structures and promotes disease progression. These processes underscore the need for novel therapeutic strategies targeting oxidative stress and neuroinflammation to alleviate the burden of VaD. The research on antioxidants and anti-inflammatory agents, both in the preclinical and clinical stages, shows promise in mitigating the effects of these pathological mechanisms. However, translating these findings into clinical therapies has proven challenging due to the complexity of VaD’s pathology, which involves both vascular and neurodegenerative components. The limited success of current therapies highlights the need for innovative approaches that simultaneously target multiple aspects of the disease process.

## Data Availability

No data were used for this study.
